# Three-port single-intercostal versus uniportal thoracoscopic segmentectomy for the treatment of lung cancer: a propensity score matching analysis

**DOI:** 10.1186/s12957-022-02626-x

**Published:** 2022-06-04

**Authors:** Keyi Sun, Zixiang Wu, Qi Wang, Ming Wu

**Affiliations:** Department of Thoracic Surgery, the Second Affiliated Hospital, Zhejiang University School of Medicine, No. 88 Jiefang Road, Hangzhou City, Zhejiang Province China

**Keywords:** Video-assisted thoracoscopic surgery, Segmentectomy, Three-port single-intercostal, Single-port

## Abstract

**Background:**

The purpose of this retrospective study was to demonstrate the safety and feasibility of three-port single-intercostal video-assisted thoracoscopic surgery (SIC-VATS) segmentectomy compared to uniportal video-assisted thoracoscopic surgery (UVATS) segmentectomy.

**Methods:**

We included 544 patients diagnosed with cT1N0M0 non-small-cell lung cancer (NSCLC) who underwent thoracoscopic segmentectomy between January 2020 and August 2021, including 147 and 397 patients who underwent three-port SIC-VATS and UVATS, respectively. After incorporating preoperative clinical variables, we compared surgical outcomes and perioperative indicators between the two groups by propensity score matching analysis.

**Results:**

After 1:1 propensity score matching, each group comprised 143 patients with no significant differences in baseline demographics and characteristics. There was no significant difference in operative time (*p* = 0.469), blood loss (*p* = 0.501), number of dissected lymph nodes (*p* = 0.228), dwell time of the main chest drain (*p* = 0.065), hospital stay (*p* = 0.243), or major complication rate (*p* = 0.295) between the three-port SIC-VATS and UVATS groups.

**Conclusions:**

The three-port SIC-VATS was as safe and feasible as UVATS for patients who are diagnosed with early-stage NSCLC.

**Supplementary Information:**

The online version contains supplementary material available at 10.1186/s12957-022-02626-x.

## Introduction

The earliest reports of video-assisted thoracic surgery (VATS) were published in the early 1990s. Over the past two decades, VATS has been recognized as a better technique than conventional thoracotomy due to the reduced risk of postoperative complications and shorter hospital stays [1, 2]. In recent years, early-stage non-small-cell lung cancer (NSCLC) has been treated via VATS in most centers [3]. In 1995, a prospective randomized controlled trial performed by the Lung Cancer Study Group (LCSG) concluded that the risk of local recurrence was higher among patients who underwent sublobar resection than among those who underwent lobectomy [4]. Therefore, lobectomy was considered the standard procedure for early-stage NSCLC. However, recent studies have found that there is no significant difference between the oncological outcomes of sublobar resection and lobectomy in patients with early-stage NSCLC [5–7].

Most surgeons use the multiple intercostal (MIC) approach during conventional VATS to complete various types of resections. However, due to injuries to multiple intercostal nerves, some patients still complain about pain in the chest wall at the surgical site several years after surgery [8]. Uniportal VATS (UVATS) was initially performed in a pulmonary lobectomy by Gonzalez et al. in 2011 [9]. Since then, the use of UVATS has expanded to involve many types of lung cancer resections. In addition to fewer injuries to the intercostal nerves [10], an advantage of the UVATS approach is that it uses the same angle of view as thoracotomy, which may be beneficial to dissection of the segmental vessel [11]. In 2014, some surgeons in our department adopted an improved technique, three-port single-intercostal VATS (SIC-VATS), to complete lung resection [12, 13]. This improved procedure can reduce the risk of injuries to the intercostal nerves and provide flexible viewing angles for lung resections and lymphadenectomy. However, no research has compared UVATS with three-port SIC-VATS. Therefore, it remains important to demonstrate the safety and feasibility of three-port SIC-VATS versus UVATS in patients with early-stage NSCLC.

## Material and methods

### Patients

From January 2020 to August 2021, we retrospectively collected data from electronic medical records and office clinical records on 544 patients who were treated with three-port SIC-VATS or UVATS segmentectomy. Clinical data were available for all the patients. The inclusion criteria were clinical stage T1N0M0 lung cancer with a single lesion less than 2 cm in diameter and ≥ 50% ground-glass appearance on computed tomography (CT) without a previous history of malignant tumors. All surgeries in this study were performed by three senior surgeons with extensive experience in thoracoscopic surgery to minimize surgeon-related bias. All the patients underwent a preoperative examination, which included high-resolution, thin-section CT of the chest, pulmonary function tests, and cardiologic assessments. The patients who were suspected of having distant metastasis were further subjected to brain magnetic resonance imaging, a bone scan, or positron emission tomography-CT. Pathological staging was performed according to the eighth edition of the American Joint Committee on Cancer staging manual [14]. Our hospital’s Research Ethics Committee approved this retrospective study.

### Surgical procedures

All patients were placed in the lateral decubitus position with the arms extended to 90°, and a soft cushion was placed under the patients to maximize the intercostal spaces to protect the intercostal nerve. During the operation, surgeons required a 10-mm, 30° electronic thoracoscope and several thoracoscopy instruments. Soft plastic incision protectors were routinely used without spreading the ribs. Most of the dissections were performed with energy devices, such as endoscopic electrocoagulation hooks and ultrasonic scalpels. All the specimens were tested by rapid pathological examination during the operation. Systemic lymph node sampling was performed when the rapid pathological examination suggested adenocarcinoma in situ (AIS) or minimally invasive adenocarcinoma (MIA). Systemic mediastinal lymphadenectomy was performed when the patients were diagnosed with invasive lung cancer. When the sampled lymph nodes were confirmed to be metastatic, the patients received pulmonary lobectomy.

**Fig. 1:**
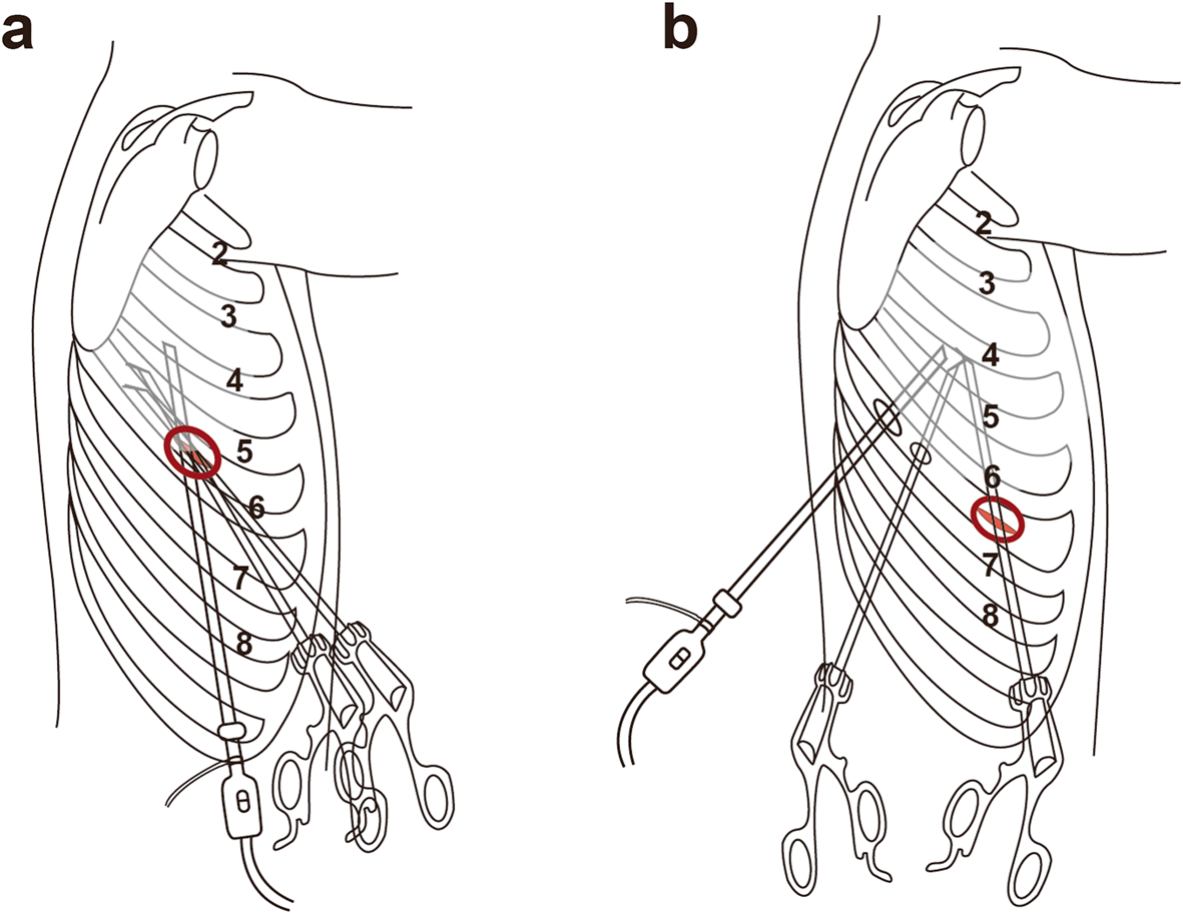
**a** The single port was located at the fifth or sixth intercostal space; **b** the three ports were located at the single sixth or seventh intercostal space

**Fig. 2:**
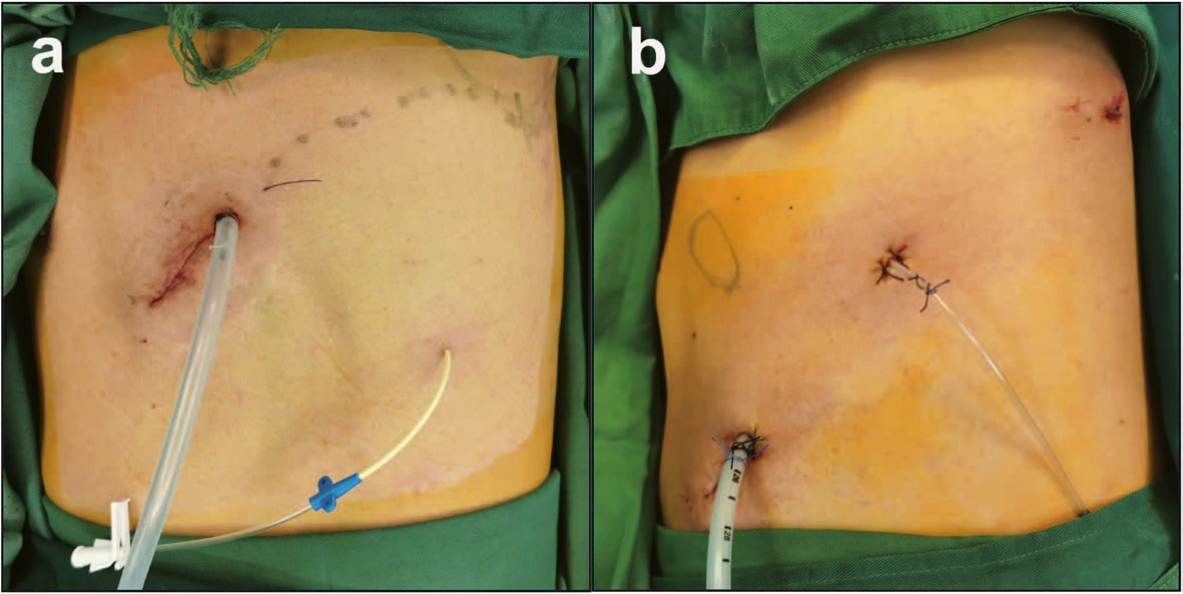
**a** Drainage tubes in UVATS; **b** drainage tubes in three-port SIC-VATS

For UVATS, the single port (4 cm) was located at the fifth or sixth intercostal space on the anterior axillary line (Fig. 1a). Under the guidance of thoracoscopy, surgeons dissected the pulmonary blood vessels and bronchi with endoscopic staplers. The inflation-deflation technique was used to identify an intersegmental plane. The lung parenchyma was resected along the junction of the expanded and atrophic lung tissue with a mechanical stapler. When the boundary was not obvious after inflation, the veins were used to locate the intersegmental plane. According to the location of the lesion, lymph nodes were systematically sampled from groups 2R, 4R, 7, 8, and 9 or groups 4L, 5, 6, 7, 8, and 9. The main chest tube (22 Fr) connected to the negative pressure drainage bottle was inserted through the port site. Then, a thin tube (14-G) was placed in the intercostal space below the wound (Fig. 2a).

We previously described the three-port SIC-VATS technique [12, 13]. Three ports, a camera port (1 cm), an auxiliary operating port (0.5 cm), and a primary port (2–3 cm), were located in the single sixth or seventh intercostal space at the posterior axillary line, midaxillary line, and anterior axillary line, respectively (Fig. 1b). Segment resection and lymph node dissection were performed in a similar manner as in UVATS. The main chest tube (22 Fr) and the assistant chest tube (8 Fr) were inserted through the utility port and secondary work port, respectively (Fig. 2b).

### Postoperative courses

The 22 Fr chest tube was removed when no obvious gas overflow was found, and plain radiography of the chest showed sufficient lung expansion. When the daily drainage was less than 200 ml, the fine chest tube was removed. Then, the patient was discharged after being assessed by senior doctors if there were no postoperative complications.

### Statistical analysis

To control for potential selection bias, we adopted 1:1 propensity score matching. Propensity scores were calculated for each patient with a logistic regression based on the following nine variables: age, sex, forced expiratory volume in 1 s (FEV1), body mass index (BMI), smoking history, tumor size, pathology, location, and pathologic stage. The match tolerance was 0.02 with the nearest neighbor matching method.

Statistical data are routinely presented as the mean (± standard deviation, 95% confidence interval). Categorical variables are expressed as percentages and proportions, and nonnormally distributed variables are summarized as medians (ranges). Nonnormally distributed data and normally distributed data were evaluated using the Mann–Whitney *U* test and Student’s *t* test, respectively. The Pearson *χ*^2^ test or Fisher’s exact test was performed to analyze categorical variables. Data were analyzed using SPSS version 25.0. When the *p* value < 0.05, the difference was considered significant.

## Results

**Fig. 3:**
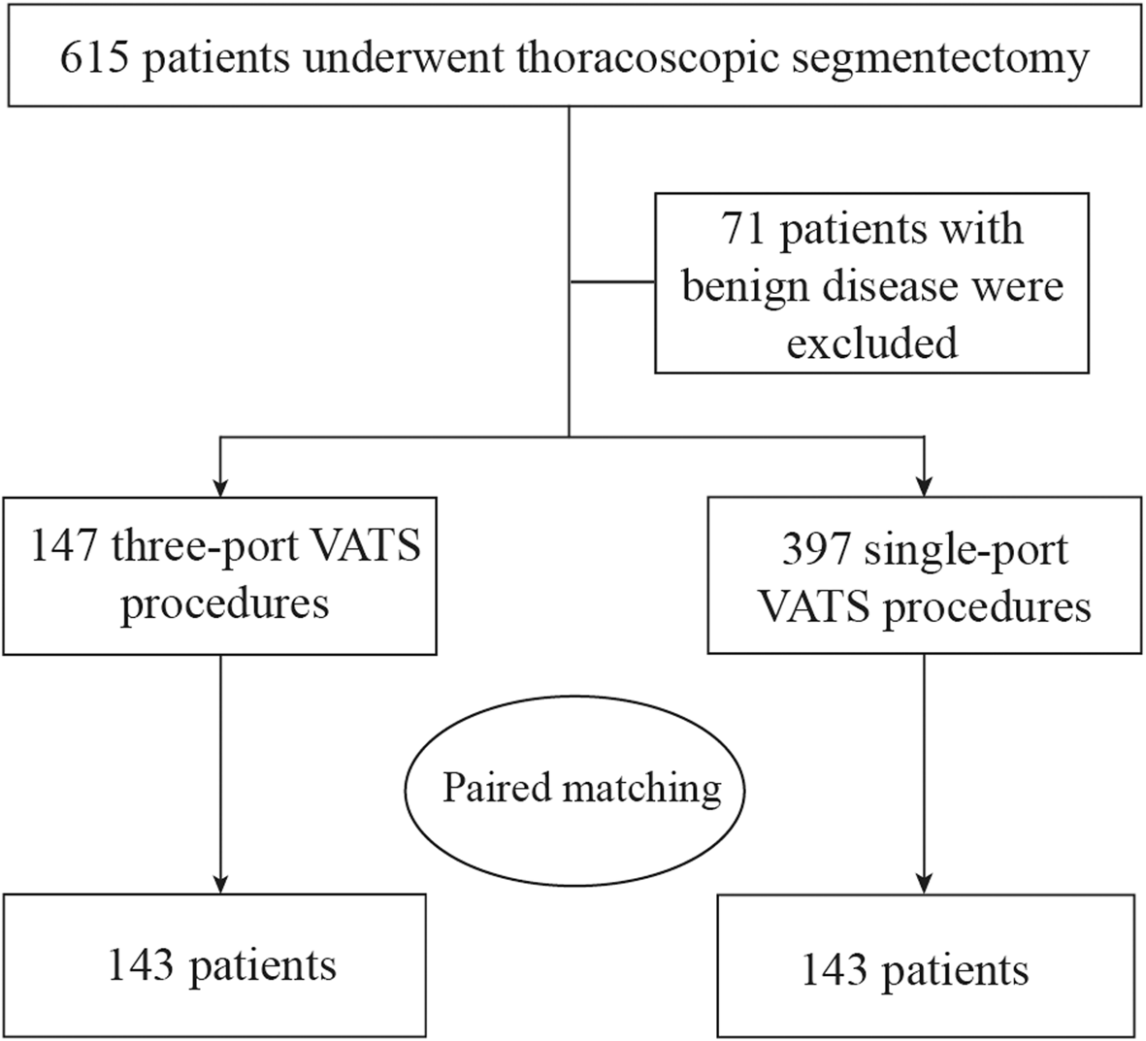
Flowchart summarizing patient enrollment

We included 544 patients with cT1N0M0 NSCLC who underwent thoracoscopic segmentectomy between January 2020 and August 2021, including 147 and 397 who underwent three-port SIC-VATS and UVATS, respectively (Fig. 3). Conversion to open thoracotomy was allowed. Finally, a total of 143 closely matched pairs were included in this study. Table 1 shows the patients’ baseline demographic data and clinical characteristics before and after matching. After propensity score matching, the baseline clinical variables were well balanced between the two groups.

**Table 1:** Baseline demographics and characteristics of the patients before and after matching

	All patients		*p* value	Propensity score-matched patients		*p* value
	Three-port (*n* = 147)	Single-port (*n* = 397)		Three-port (*n* = 143)	Single-port (*n* = 143)	
Age (years)	55.2 (13.7)	53.2 (12.6)	0.108	54.8 (13.7)	54.6 (11.9)	0.894
Sex			< 0.001			0.884
Male	30 (20.4%)	146 (36.8%)		30 (21.0%)	29 (20.3%)	
Female	117 (80.3%)	251 (63.2%)		113 (79.0%)	114 (79.7%)	
FEV1 (L)	2.5 (0.7)	2.6 (0.7)	0.084	2.5 (0.7)	2.5 (0.6)	0.552
Smoking history			0.048			0.557
Current or ever	16 (10.9%)	71 (17.9%)		16 (11.2%)	13 (9.1%)	
Never	131 (89.1%)	326 (82.1%)		127 (88.8%)	130 (90.9%)	
BMI (kg/m^2^)	23.1 (3.1)	23.0 (2.9)	0.736	23.1 (3.1)	23.2 (2.9)	0.702
Tumor size (cm)	1.1 (0.5)	1.1 (0.4)	0.849	1.1 (0.5)	1.1 (0.4)	0.374
Location			0.380			0.591
LUL	44 (29.9%)	96 (24.2%)		41 (28.7%)	38 (26.6%)	
LLL	19 (12.9%)	55 (13.9%)		19 (13.3%)	24 (16.7%)	
RUL	56 (38.1%)	147 (37.0%)		56 (39.2%)	48 (33.6%)	
RML	0 (0%)	0 (0%)		0 (0%)	0 (0%)	
RLL	28 (19.0%)	99 (24.9%)		27 (18.9%)	33 (23.1%)	
Pathology			0.397			0.678
AIS	30 (20.4%)	103 (25.9%)		30 (21.0%)	31 (21.7%)	
MIA	43 (29.2%)	112 (28.2%)		43 (30.1%)	49 (34.3%)	
IA	74(43.5%)	182(45.8%)		70 (49.0%)	63 (44.1%)	
Stage			0.390			0.689
IA1	40 (27.2%)	86 (21.7%)		37 (25.9%)	32 (22.4%)	
IA2	34 (23.1%)	96 (24.2%)		33 (23.1%)	31 (21.7%)	

Data are presented as the *n* (%) or the mean (SD). *LUL* left upper lobe, *LLL* Left lower lobe, *RUL* Right upper lobe, *RLL* Right lower lobe, *AIS* Adenocarcinoma in situ, *MIA* Minimally invasive adenocarcinoma, *IA* Invasive adenocarcinoma

The types of resected lung segments are presented in Table 2. Most cases were apical segments, apical posterior segments, and superior segments, followed by left lingual segments and upper divisional segments. No patient underwent right middle interior or lateral segmentectomy.

**Table 2:** Thoracoscopic segmentectomy in both groups

Segmentectomy	Three-port (*n* = 143)	Single-port (*n* = 143)
LUL		
Apical posterior segmentectomy	22	16
Anterior segmentectomy	2	5
Lingual segmentectomy	10	8
Upper divisional segmentectomy	8	11
LLL		
Superior segmentectomy	13	16
Basal segmentectomy	7	8
RUL		
Apical segmentectomy	10	24
Posterior segmentectomy	13	9
Apical posterior segmentectomy	22	4
Anterior segmentectomy	11	12
RLL		
Superior segmentectomy	22	11
Basal segmentectomy	7	23

*LUL*, Left upper lobe, *LLL* Left lower lobe, *RUL* Right upper lobe, *RLL* Right lower lobe

The operative details and perioperative data of both propensity score-matched groups are presented in Table 3. The mean operative time of three-port SIC-VATS segmentectomy was 114.0 min (38.0; 95% CI: 107.7–120.3), which was shorter than that of UVATS (116.9 min (29.3; 95% CI: 112.1–122.8)). However, there were no significant differences in operative time between the two groups (*p* = 0.469). In addition, no significant difference was found in blood loss, the number of lymph node stations harvested, the total number of lymph nodes removed, the indwelling time of the main chest drain (22 Fr), hospital stay, or the major complication rate between the two matched groups. Conversion to thoracotomy occurred in one patient in the three-port SIC-VATS group due to anatomical variants and in one patient in the UVATS group due to vessel injury. No deaths occurred during the perioperative period in either group.

**Table 3:** Perioperative outcomes of the patients in both groups

	Three-port (*n* = 143)	Single-port (*n* = 143)	*p* value
Operative time (min)	114.0 (38.0, 107.7–120.3)	116.9 (29.3, 112.1–122.8)	0.469
Blood loss (mL)^a^	31 (10–140)	32 (10–1000)	0.501
Conversion	1 (0.7%)	1 (0.7%)	1.000
LN assessment			
Total LNs removed	11.9 (5.3, 11.0–12.8)	12.7 (5.7, 11.8–13.6)	0.228
Total LN stations removed	6.7 (2.2, 6.3–7.0)	7.0 (2.3, 6.7–7.4)	0.174
Chest tube removal (22-Fr) (PODs)^a^	1 (1–13)	1 (1–8)	0.065
Hospitalization after the operation (days)	3.6 (1.9, 3.2–3.9)	3.3 (1.3, 3.1–3.5)	0.243
Major complications			
Chylothorax	1 (0.7%)	2 (1.4%)	0.562
Atelectasis	1 (0.7%)	1 (0.7%)	1.000
Pulmonary embolism	1 (0.7%)	0 (0.0%)	0.316
Empyema	0 (0.0%)	0 (0.0%)	1.000
Atrial fibrillation	8 (5.6%)	4 (2.8%)	0.238
Prolonged air leak^b^	5 (3.5%)	3 (2.1%)	0.473
Reoperation	0 (0.0%)	0 (0.0%)	1.000
Total	15 (10.5%)	10 (7.0%)	0.295
Mortality	0 (0.0%)	0 (0.0%)	1.000

Data are presented as the median (range) or the mean (± SD, 95% confidence interval). *LN* Lymph node, *PODs* Postoperative days. ^a^Skewed distribution: the Mann–Whitney *U* test was applied. ^b^Prolonged air leak was defined as a sustained air leakage lasting 5 days or more

Table 4 describes whether the surgical outcomes (operative time and blood loss) varied with the demographic and clinical variables (age, sex, lesion location, and tumor size) for the two matched groups. We found that the between-group and within-group differences were not statistically significant.

**Table 4:** Comparison of clinical variables and perioperative outcomes in the two groups

	Operative time (min)		*p* value	Blood loss (mL)^a^		*p* value
	Three-port (*n* = 143)	Single-port (*n* = 143)		Three-port (*n* = 143)	Single-port (*n* = 143)	
Age (years)						
< 55	111.5 (31.4)	119.2 (31.0)	0.151	30 (10 –125)	34 (10 –50)	0.204
≥ 55	116.4 (43.4)	114.9 (28.0)	0.809	35 (10 –140)	32 (10 –1000)	0.840
*p* value	0.444	0.384		0.289	0.913	
Sex						
Male	118.5 (48.0)	117.4 (31.3)	0.914	31 (10 –60)	36 (10 –1000)	0.305
Female	112.8 (35.0)	116.8 (29.0)	0.349	31 (10 –140)	32 (10 –100)	0.757
*p* value	0.465	0.926		0.678	0.152	
Location						
Left upper lobe	111.1 (27.7)	117.5 (29.2)	0.332	32 (10 –140)	36 (10 –1000)	0.403
Left lower lobe	128.3 (48.7)	120.3 (32.0)	0.521	29 (10 –54)	35 (12 –100)	0.321
*p* value	0.164	0.718		0.440	0.854	
Right upper lobe	107.7 (41.4)	114.4 (27.1)	0.343	29 (12 –120)	35 (10 –50)	0.659
Right lower lobe	121.4 (33.2)	117.6 (31.9)	0.648	34 (10 –125)	30 (10 –50)	0.426
*p* value	0.138	0.629		0.480	0.647	
Tumor size (cm)						
≤ 1	113.4 (37.7)	119.4 (30.5)	0.254	31 (10 –125)	35 (10 –52)	0.426
> 1	115.0 (38.5)	113.2 (27.5)	0.789	32 (10 –140)	32 (10 –1000)	0.775
*p* value	0.811	0.222		0.969	0.956	

Data are presented as the median (range) or the mean (SD). ^a^Skewed distribution: the Mann–Whitney *U* test was applied

## Discussion

To our knowledge, this is one of the first studies to compare the perioperative outcomes of three-port SIC-VATS segmentectomy versus UVATS. In this retrospective study, propensity score matching was applied to minimize the influence of potential selection bias and confounding bias between the two groups. In general, the two surgical techniques were comparable.

The results of the only randomized controlled trial published to date on this issue established that lobectomy is the first choice for the treatment of early-stage NSCLC [4]. However, the inclusion criteria of this trial (tumor ≤ 3 cm) and the surgical selection (wedge resection accounted for 32.8% of the patients in the sublobar resection group) may affect the reliability of the results. In addition, there is a large difference in the surgical technique between that time and the present time. In 2011, Schuchert et al. [5] found that the 5-year recurrence rate and 5-year overall survival rate of wedge resection, segmentectomy, and lobectomy for early-stage NSCLC with a tumor diameter less than 1 cm were similar. In 2015, Yano et al. [6] published the results of a multicenter retrospective study, which showed that in 1737 patients who underwent sublobar resection (segmentectomy accounted for 63% of the patients), the 5-year overall survival rate reached 94%. A recent meta-analysis suggested that segmentectomy might be comparable to lobectomy for stage IA NSCLC with a tumor size < 2 cm [15]. With the popularity of low-dose spiral CT, an increasing number of pulmonary nodules have been found, and most medical centers have generally adopted sublobar resection to treat early-stage NSCLC. Compared with wedge resection, segmentectomy can ensure a sufficient distance of the lesion from the resection edge, thereby reducing the recurrence rate and improving long-term survival [16]. At present, segmentectomy has received increasing attention from surgeons for the treatment of early-stage NSCLC.

Data from propensity score-matched cohorts confirmed that the operative time was comparable between three-port SIC-VATS and UVATS. Although this trend was different from that described in other reports, in which UVATS was associated with a shorter operation time [17, 18], our result was consistent with those of some previous studies [19, 20]. In the three-port SIC-VATS group, the mean operative time for lesions in the left lower lobe was 128.3 min, which contrasted with a mean operative time of 111.1 min for lesions in the left upper lobe. Although there appeared to be an obvious difference, it was not statistically significant (*p* = 0.164). The possible reasons for this finding are unclear, but they might be due to the narrow visual field when a tumor is located in the lower lobe of the lung, which makes individual dissection of the segmental vessels and bronchi difficult. The greatest advantage of UVATS is that it provides a similar angle of view as that of thoracotomy regardless of tumor location. Therefore, the operative time is not affected by the tumor location in UVATS. Overall, our study does not support a shorter operative time in UVATS.

Adequate dissection of the lymph nodes is one of the critical steps to maintain consistent-quality surgical procedures. The guidelines published by the National Comprehensive Cancer Network recommend systematic mediastinal lymph node dissection in lobectomy [21]. However, it is still controversial whether systematic mediastinal lymph node dissection is necessary in segmentectomy. Resection of at least 6 nodes is recommended according to guidelines published by the European Society of Thoracic Surgeons, as this guarantees a proper pathological classification [22]. In our research, the mean numbers of dissected lymph nodes were 11.9 and 12.7 for three-port SIC-VATS and UVATS, respectively, which was consistent with previous studies [20, 23].

In recent years, the incidence of major complications of thoracoscopic pulmonary segmentectomy has been reported to be 8–24.8% [17, 18, 24]. In our series, the complication rate was 8.2–11.6%, which was similar to the complication rate presented in previous studies. Postoperative atrial fibrillation was a common complication, occurring in 8 and 4 patients in the three-port SIC-VATS and UVATS groups, respectively. Cardioversion drugs were effective in recovering heart rhythm.

The thoracic drainage tube is one of the main causes of postoperative pain [25]. Therefore, we have made some improvements to traditional indwelling chest tube surgery. A main chest tube (22 Fr) and a thin chest tube (8 Fr and 14 G in the three-port and uniport groups, respectively) were placed in the operation port. Compared with the main chest tube, the thin chest tube causes less pain. In our research, most patients had the main chest tube removed on the first day after surgery. This improved technology can alleviate patients’ pain and help them become ambulatory as soon as possible.

Many thoracic surgeons still perform traditional three-port operations. However, it is more difficult to convert from traditional three-port VATS to UVATS due to the change in operation angle and the interference of instruments. We offer these thoracic surgeons another surgical option. Compared with conventional multiport VATS, three-port SIC-VATS theoretically has the advantage of injuring only one intercostal nerve. Furthermore, SIC-VATS requires no extra instruments and still has a flexible operating angle. In our experience, this helps surgeons navigate the learning curve safely. This should be an advantage of SIC-VATS. Uniportal VATS has higher requirements for the surgical assistant because he or she needs to adjust the lens frequently and avoid instrument interference simultaneously. During the entire operation, the assistant has to keep his or her arms raised to hold the thoracoscope. Moreover, as the anatomical location changes, the positions of the surgeon and assistant also need to change. In addition, due to the limitation of a single operating port, uniportal VATS may require more angled staplers than three-port SIC-VATS, which may increase costs.

There are a number of study limitations that must be considered. First, the choice of SIC-VATS or UVATS depended on surgeon preference. Although we generated balanced groups according to several variables by propensity score matching, potential selection bias could not be completely eliminated due to the retrospective nature of this study. Second, although all procedures were performed by three well-experienced surgeons, there was inevitable surgical heterogeneity among them. Third, due to the limitation of the retrospective design, postoperative management in the two groups was different, and the pain analysis of SIC-VATS versus UVATS could not be evaluated in this study. Therefore, prospective, multicenter, randomized studies are needed to verify these conclusions.

## Conclusions

In this propensity-matched analysis, we demonstrated that three-port SIC-VATS segmentectomy and UVATS segmentectomy have comparable perioperative outcomes. In summary, the three-port SIC-VATS was as safe and feasible as UVATS for patients who are diagnosed with early-stage NSCLC.

## Supplementary Information

**Additional file 1.**

## Data Availability

All data generated and analyzed during this study are included in this published article and its supplementary information files.

## Abbreviations

CI: : Confidence interval
FEV1: : Forced expiratory volume in the first second
NSCLC: : Non-small-cell lung cancer
POD: : Postoperative day
MIC: : Multiple intercostal
SIC: : Single intercostal
VATS: : Video-assisted thoracoscopic surgery
LCSG: : Lung Cancer Study Group
CT: : Computed tomography
AIS: : Adenocarcinoma in situ
MIA: : Minimally invasive adenocarcinoma
IA: : Invasive adenocarcinoma

## References

[1] Paul S, Altorki NK, Sheng S, Lee PC, Harpole DH, Onaitis MW (2010). Thoracoscopic lobectomy is associated with lower morbidity than open lobectomy: a propensity-matched analysis from the STS database. J Thorac Cardiovasc Surg.

[2] Whitson BA, Andrade RS, Boettcher A, Bardales R, Kratzke RA, Dahlberg PS (2007). Video-assisted thoracoscopic surgery is more favorable than thoracotomy for resection of clinical stage I non-small cell lung cancer. Ann Thorac Surg.

[3] Yan TD, Black D, Bannon PG, McCaughan BC (2009). Systematic review and meta-analysis of randomized and nonrandomized trials on safety and efficacy of video-assisted thoracic surgery lobectomy for early-stage non-small-cell lung cancer. J Clin Oncol.

[4] Ginsberg RJ, Rubinstein LV (1995). Randomized trial of lobectomy versus limited resection for T1 N0 non-small cell lung cancer. Lung Cancer Study Group. Ann Thorac Surg..

[5] Schuchert MJ, Kilic A, Pennathur A, Nason KS, Wilson DO, Luketich JD (2011). Oncologic outcomes after surgical resection of subcentimeter non-small cell lung cancer. Ann Thorac Surg..

[6] Yano M, Yoshida J, Koike T, Kameyama K, Shimamoto A, Nishio W (2015). Survival of 1737 lobectomy-tolerable patients who underwent limited resection for cStage IA non-small-cell lung cancer. Eur J Cardiothorac Surg.

[7] Subramanian M, McMurry T, Meyers BF, Puri V, Kozower BD (2018). Long-term results for clinical stage IA lung cancer: comparing lobectomy and sublobar resection. Ann Thorac Surg.

[8] Rizk NP, Ghanie A, Hsu M, Bains MS, Downey RJ, Sarkaria IS (2014). A prospective trial comparing pain and quality of life measures after anatomic lung resection using thoracoscopy or thoracotomy. Ann Thorac Surg.

[9] Gonzalez D, Paradela M, Garcia J, Dela TM (2011). Single-port video-assisted thoracoscopic lobectomy. Interact Cardiovasc Thorac Surg.

[10] Jutley RS, Khalil MW, Rocco G (2005). Uniportal vs standard three-port VATS technique for spontaneous pneumothorax: comparison of post-operative pain and residual paraesthesia. Eur J Cardiothorac Surg.

[11] Rocco G, Martin-Ucar A, Passera E (2004). Uniportal VATS wedge pulmonary resections. Ann Thorac Surg.

[12] Wang L, Pan S, Wu M (2017). Video-assisted thoracoscopic sleeve lobectomy via a single intercostal space three-port approach: case report. Medicine (Baltimore).

[13] Wu Z, Fang S, Wang Q, Wu C, Zhan T, Wu M (2018). Patient-controlled paravertebral block for video-assisted thoracic surgery: a randomized trial. Ann Thorac Surg.

[14] Detterbeck FC, Boffa DJ, Kim AW, Tanoue LT (2017). The eighth edition lung cancer stage classification. Chest.

[15] Winckelmans T, Decaluwé H, De Leyn P, Van Raemdonck D (2020). Segmentectomy or lobectomy for early-stage non-small-cell lung cancer: a systematic review and meta-analysis. Eur J Cardiothorac Surg.

[16] Zhao M, Lu T, Huang Y, Yin J, Jiang T, Li M (2019). Survival and long-term cause-specific mortality associated with stage IA lung adenocarcinoma after wedge resection vs. segmentectomy: a population-based propensity score matching and competing risk analysis. Front Oncol..

[17] Wang BY, Liu CY, Hsu PK, Shih CS, Liu CC (2015). Single-incision versus multiple-incision thoracoscopic lobectomy and segmentectomy: a propensity-matched analysis. Ann Surg.

[18] Liu CC, Shih CS, Pennarun N, Cheng CT (2016). Transition from a multiport technique to a single-port technique for lung cancer surgery: is lymph node dissection inferior using the single-port technique?†. Eur J Cardiothorac Surg.

[19] Shih C-S, Liu C-C, Liu Z-Y, Pennarun N, Cheng C-T. Comparing the postoperative outcomes of video-assisted thoracoscopic surgery (VATS) segmentectomy using a multi-port technique versus a single-port technique for primary lung cancer. Journal of Thoracic Disease. 2016:S287-S94 10.3978/j.issn.2072-1439.2016.01.78 PMC4783730 27014476

[20] Xie D, Wu J, Hu X, Gonzalez-Rivas D, She Y, Chen Q (2021). Uniportal versus multiportal video-assisted thoracoscopic surgery does not compromise the outcome of segmentectomy. Eur J Cardiothorac Surg.

[21] National Comprehensive Cancer Network. Non-small cell lung cancer (version 5. 2021). [Available from: https://www.nccn.org/professionals/physician_gls/pdf/nscl.pdf.

[22] Lardinois D, De Leyn P, Van Schil P, Porta RR, Waller D, Passlick B (2006). ESTS guidelines for intraoperative lymph node staging in non-small cell lung cancer. Eur J Cardiothorac Surg.

[23] Han KN, Kim HK, Choi YH (2016). Comparison of single port versus multiport thoracoscopic segmentectomy. J Thorac Dis.

[24] Donahue JM, Morse CR, Wigle DA, Allen MS, Nichols FC, Shen KR (2012). Oncologic efficacy of anatomic segmentectomy in stage IA lung cancer patients with T1a tumors. Ann Thorac Surg..

[25] McElnay PJ, Molyneux M, Krishnadas R, Batchelor TJ, West D, Casali G (2015). Pain and recovery are comparable after either uniportal or multiport video-assisted thoracoscopic lobectomy: an observation study. Eur J Cardiothorac Surg.

